# Low Area Specific Resistance La-Doped Bi_2_O_3_ Nanocomposite Thin Film Cathodes for Solid Oxide Fuel Cell Applications

**DOI:** 10.1021/acs.nanolett.4c03679

**Published:** 2024-11-26

**Authors:** Adam J. Lovett, Matthew P. Wells, Yizhi Zhang, Jiawei Song, Thomas S. Miller, Haiyan Wang, Judith L. MacManus-Driscoll

**Affiliations:** † Department of Materials Science and Metallurgy, University of Cambridge, 27 Charles Babbage Road, Cambridge, United Kingdom, CB3 0FS; ‡ Electrochemical Innovation Lab, Department of Chemical Engineering, University College London, Torrington Place, London, United Kingdom, WC1E 7JE; § School of Materials Engineering, Purdue University, 701 West Stadium Avenue, West Lafayette, Indiana 47907-2045, United States

**Keywords:** energy materials, ion conductivity, nanocomposite, solid oxide fuel cell, epitaxial thin film, bismuth oxide

## Abstract

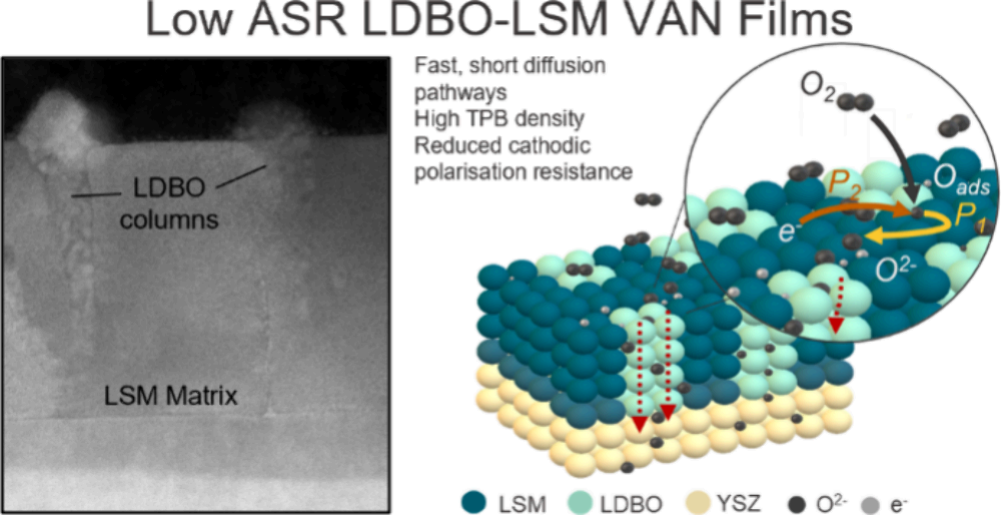

In the context of solid oxide fuel cells (SOFCs), vertically aligned nanocomposite (VAN) thin films have emerged as a leading material type to overcome performance limitations in cathodes. Such VAN films combine conventional cathodes like La_*x*_Sr_1–*x*_Co_*y*_Fe_1–*y*_O_3_ (LSCF) and La_1–x_Sr_*x*_MnO_3_ (LSM) together with highly O^2–^ ionic conducting materials including yttria-stabilized zirconia (YSZ) or doped CeO_2_. Next-generation SOFCs will benefit from the exceptionally high ionic conductivity (1 S cm^–1^ at 730 °C) of Bi_2_O_3_-based materials. Therefore, an opportunity exists to develop Bi_2_O_3_-based VAN cathodes. Herein, we present the first growth and characterization of a Bi_2_O_3_-based VAN cathode, containing epitaxial La-doped Bi_2_O_3_ (LDBO) columns embedded in a LSM matrix. Our novel VANs exhibit low area specific resistance (ASR) (8.3 Ω cm^2^ at 625 °C), representing ∼3 orders of magnitude reduction compared to planar LSM. Therefore, by demonstrating a high-performance Bi_2_O_3_-based cathode, this work provides an important foundation for future Bi_2_O_3_-based VAN SOFCs.

In solid oxide fuel cells (SOFCs), composite cathodes formed by combining a superionic conductor with an electrocatalytically active cathode material can overcome the intrinsically low oxide ion conductivity of conventional cathode materials such as La_0.8_Sr_0.2_MnO_3_ (LSM). In recent years vertically aligned nanocomposite (VAN) thin films, a specific type of nanostructured composite, have emerged as a leading class of materials for SOFC electrodes.^1−7^ Typically, VAN films are characterized as self-assembled nanostructures in which columns of one material are embedded in a matrix of another. These unique structures enable a wide range of enhanced, tunable performance characteristics resulting from the high density of vertical interfaces between the two phases and user-chosen epitaxial orientation. In particular, SOFC VAN films have yielded enhanced oxygen reduction reaction kinetics,^8^ enhanced ionic conductivity,^9^ and improved long-term stability.^1^ To date, VAN films for SOFC applications have primarily been based on yttrium-stabilized zirconia (YSZ) or doped CeO_2_. Therefore, an opportunity exists to expand and explore new, novel material combinations for next-generation, low-temperature SOFC devices.

As a potential O^2–^ superionic conductor in a SOFC device, doped Bi_2_O_3_ phases with the face centered cubic (fcc) defect-fluorite structure (δ-Bi_2_O_3_) are naturally attractive due to their intrinsically high ionic conductivities (up to 1 S cm^–1^ at 730 °C for undoped δ-Bi_2_O_3_), 2 orders of magnitude higher than widely used YSZ at corresponding temperatures.^10^ Indeed, bulk composite cathodes based on La_0.85_Sr_0.15_MnO_3±δ_ (LSM) and (Bi_0.8_Er_0.2_)_2_O_3_ (ESB) have shown exceptional cathodic performance, characterized by very low area specific resistance (ASR) of 0.1 Ω cm^2^ and a corresponding peak power density of 1.2 W cm^–2^ at only 600 °C.^11^ In thin films, the defect-fluorite structure has been successfully stabilized with epitaxial templating in both planar^12−14^ and superlattice films.^15,16^ However, for planar epitaxial films, substrate strain relaxation occurs above a few 10s of nm,^17^ which limits the achievable thickness and structural robustness of the δ-Bi_2_O_3_, particularly during thermal treatment.^12^ Additionally, superlattice structures utilize extremely thin (<3 nm) layers of δ-Bi_2_O_3_ grown between carefully chosen supporting layers,^15,16^ consequently restricting the combination of phases that can be paired. Crucially, in a practical SOFC device it is a requirement that the ion transport be out-of-the-plane of the film to enable ionic transport between top and bottom electrodes.^18^ However, in superlattice films the ionic conducting channels are confined within the plane of the film.^18^ Hence, while superlattices of SOFC-relevant phases are of interest academically, they are rarely of practical use in SOFC devices. Therefore, by moving towards a structure that both enables epitaxial templating and contains out-of-plane ionic transport, such as the aforementioned VAN films, a platform to build a practical Bi_2_O_3_-containing SOFC device would be enabled. Such a SOFC device would benefit from the intrinsically high ionic conductivities of Bi_2_O_3_ phases, thus offering a route to the development of future low-temperature (<600 °C) SOFC devices.

We recently demonstrated that the δ-Bi_2_O_3_ phase can be epitaxially templated in a Dy-stabilized Bi_2_O_3_–DyMnO_3_ (DSB-DMO) VAN.^10^ This VAN structure comprises DSB columns embedded in a matrix of DMO. Here, the structural relationship between DMO and DSB is integral to achieving epitaxial templating. The DMO matrix forms a strained tetragonal phase (*a* = *b* = 5.53 Å, *c* = 7.65 Å^10^) rather than the bulk orthorhombic GdFeO_3_ phase (*a* = 5.280 Å, *b* = 5.832 Å, *c* = 7.381 Å^19^) that minimizes lattice mismatch (∼2%) with the Dy-doped δ-Bi_2_O_3_ structure (*a* = 5.418 Å^10^). Thus, the DMO matrix acts as a scaffold to stabilize the DSB columnar phase, crucially, beyond the critical thickness (typically ∼20 nm) in planar and superlattice films.^17^ Consequently, the DSB-DMO VAN films benefit from the intrinsically high ionic conductivity of DSB (10^–3^ S cm^–1^ at 500 °C) in a device architecture where the DSB oxide ion conduction channels are perpendicular to the substrate, thus satisfying the SOFC out-of-plane ionic transport requirement.

A natural progression of this work is the replacement of DMO with a SOFC-relevant material to form a composite cathode. The logical choice is La_0.8_Sr_0.2_MnO_3_ (LSM), an established SOFC cathode that, as previously mentioned, has been reported in bulk LSM-doped Bi_2_O_3_ composite cathodes.^20−22^ Structurally, undoped LaMnO_3_ adopts the same orthorhombic GdFeO_3_ perovskite structure as bulk DMO.^19,23^ Upon doping with strontium, bulk LSM forms the rhombohedral crystal structure (*a* = 5.52 Å, *c* = 13.35 Å^24^) which contains a pseudocubic unit cell comprised of perovskite cubes with lattice parameter *a*_pc[100]_ = 3.89 Å in the [100] direction or *a*_pc[110]_ = 5.52 Å in the [110] direction.^25,26^ Hence, due to the structural similarity of the strained DMO phase in our previous VAN to the pseudocubic LSM, we hypothesized that epitaxial templating should be achievable to obtain a Bi_2_O_3_-based VAN, thus enabling a low ASR cathode for SOFC devices.

In this letter, we report the first successful growth and electrochemical characterization of a Bi_2_O_3_-based VAN thin film cathode material. In particular, we demonstrate a VAN structure comprising of La-doped Bi_2_O_3_ (LDBO) columns embedded in an LSM matrix (herein referred to as LDBO-LSB VAN) grown on (001)-oriented yttrium-stabilized zirconia (YSZ) substrates. Then, through electrochemical impedance spectroscopy (EIS), we measure low ASR values of 8.3 Ω cm^2^ at 625 °C, representing approximately 3 orders of magnitude reduction compared to planar LSM. Our results affirm that Bi_2_O_3_ phases can be successfully epitaxially templated with the VAN architecture, thus enabling a low ASR cathode for SOFC devices. In addition to the potential for lower temperature operation SOFCs, there is also potential for improved long-term stability of the cathode–electrolyte interface owing to the use of the same material, thus avoiding chemical reaction at the SOFC operational temperature. This work therefore marks an important advance in demonstrating the foundation for future Bi_2_O_3_-based VAN SOFCs.

We first investigate the structure of our LDBO-LSM VAN cathode thin films grown on YSZ (001) substrates (Figure 1). From high-resolution STEM images (Figure 1a,b), clear subtly triangular columnar structures with surface diameters of 20–50 nm are seen throughout a very dense matrix. These columns are comprised of (001)-oriented rhombohedral lanthanum-doped Bi_2_O_3_, as determined from Bi and La contrast HAADF-STEM images (Figure 1c and Supplementary Figure 1, respectively) and the indexed 2θ–ω XRD pattern (Figure 1d) (*c* = 27.686(5) Å, determined from Nelson–Riley function extrapolation). The *c* lattice parameter of rhombohedral La-doped Bi_2_O_3_ increases with decreasing lanthanum content. Since the *c* lattice parameter of our LDBO columns is lower than rhombohedral La_0.45_Bi_1.55_O_3_ (*c* = 27.6004(9) Å^27,28^) or La_0.60_Bi_1.40_O_3_ (*c* = 27.557(7) Å^29^), we extrapolate a molar fraction of La < 0.45 in the LDBO columns (assuming that strain plays no significant role).

**Figure 1:**
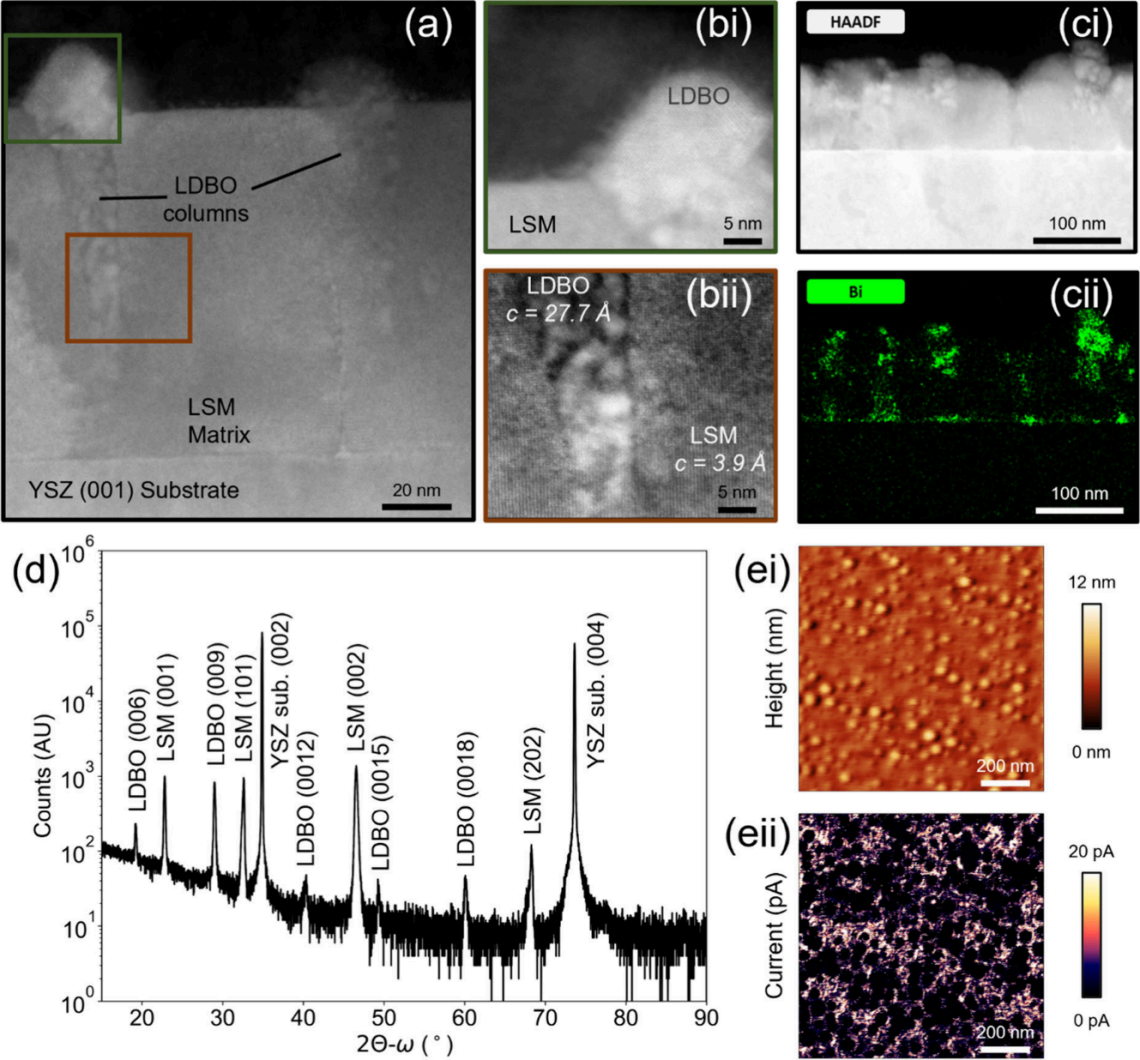
Structural characterization of LDBO-LSM VAN films: (a) high-resolution cross-sectional STEM images showing epitaxial LSM matrix and embedded LDBO columns with enlarged regions from (bi) green and (bii) brown boxes. (ci) HAADF-STEM and (cii) bismuth EDX images confirming localized Bi as part of fast ionic conducting LDBO columns. (d) XRD pattern of LDBO-LSM VAN film on YSZ (001), indicative of the epitaxial nature of both phases, i.e., very sharp LDBO (00*l*) family of reflections and both the LSM (00*l*) and LSM (*h*0*l*) *h* = *l* families. (ei) AFM height and (eii) conducting-AFM images confirming electronically insulating LDBO columns embedded in a conductive LSM matrix.

Lanthanum incorporation in the Bi_2_O_3_ lattice is not unexpected and occurs due to intermixing from the LSM matrix. Low-level intermixing is common in VAN films.^10,30,31^ Nonetheless in this case it is very beneficial, as it aids stabilization of the high ionic conducting LDBO phase, which has a 1–2 order of magnitude improved ionic conductivity over YSZ and samarium-doped CeO_2_ (SDC) at comparable temperatures in the range 400–600 °C.^32,33^ Further, at high temperatures (>∼600 °C) low La content (such as La_0.3_Bi_1.7_O_3_) LDBO has a higher ionic conductivity than ESB with the fcc δ-Bi_2_O_3_ structure.^32,34^

Next, we consider the LSM matrix. From XRD (Figure 1d), the LSM is composed of two orientations of LSM with the pseudocubic structure (*a*_pc[100]_ = 3.89 Å^25^): (001) *c* = 3.903 (1) Å and (101) *c* = 3.88 (5) Å. The presence of two orientations of LSM was also observed in previous LSM-SDC VAN cathodes grown on YSZ (001) and does not hinder electrochemical performance.^1^ Crucially, the LDBO-LSM VAN contains a high density of gas–cathode (LSM)–electrolyte (LDBO) triple phase boundaries (TPBs), which is highly desirable for a low ASR SOFC cathode.^1,3^ The TPB presence is evident from the distribution of insulating LDBO columns which are round in shape (consistent with TEM observations in Figure 1a), embedded in the highly conductive LSM matrix observed in conducting atomic force microscopy (C-AFM) studies (Figure 1e). By considering the boundaries between the conductive matrix and insulating columns observed in C-AFM (Figure 1eii), we calculate the TPB density to be 4.9 × 10^5^ cm cm^–2^, in line with previous estimates for VAN SOFC cathodes.^1^ As we will discuss later, this very high density of TPBs enables low ASR, further aided by the fast O^2–^ diffusion within the LDBO-LSM VAN cathode. Note here that the LDBO-LSM film was grown on a Nb-SrTiO_3_ (001) with the same epitaxial relationship (see Supplementary Figure S2) to facilitate electronic conductivity through the substrate for investigative purposes.

Now we focus our attention on the electrochemical properties of our LDBO-LSM films (Figure 2). First, the *p*O_2_-dependent polarization resistance at 500 °C of a Ag/LDBO-LSM/YSZ/Ag system is investigated with EIS. Nyquist plots (Figure 2a) display three separable features, well-modeled by the equivalent circuit model inset in Figure 2a: *R*_YSZ_ corresponding to the O^2–^ ionic resistance through the YSZ substrate (from EIS Arrhenius data in Figure 2c, *E*_a_ = 1.15 eV, consistent with previous literature for YSZ^35^), in series with two resistor-constant phase element units (R_1_-CPE_1_ at intermediate frequencies and R_2_-CPE_2_ at lower frequencies) corresponding to two separate oxygen reduction reaction (ORR) electrode processes (P_1_ and P_2_, respectively). The EIS equivalent circuit model for our thin films is analogous to those used for bulk LSM-Bi_2_O_3_ composite cathodes.^20−22^

**Figure 2:**
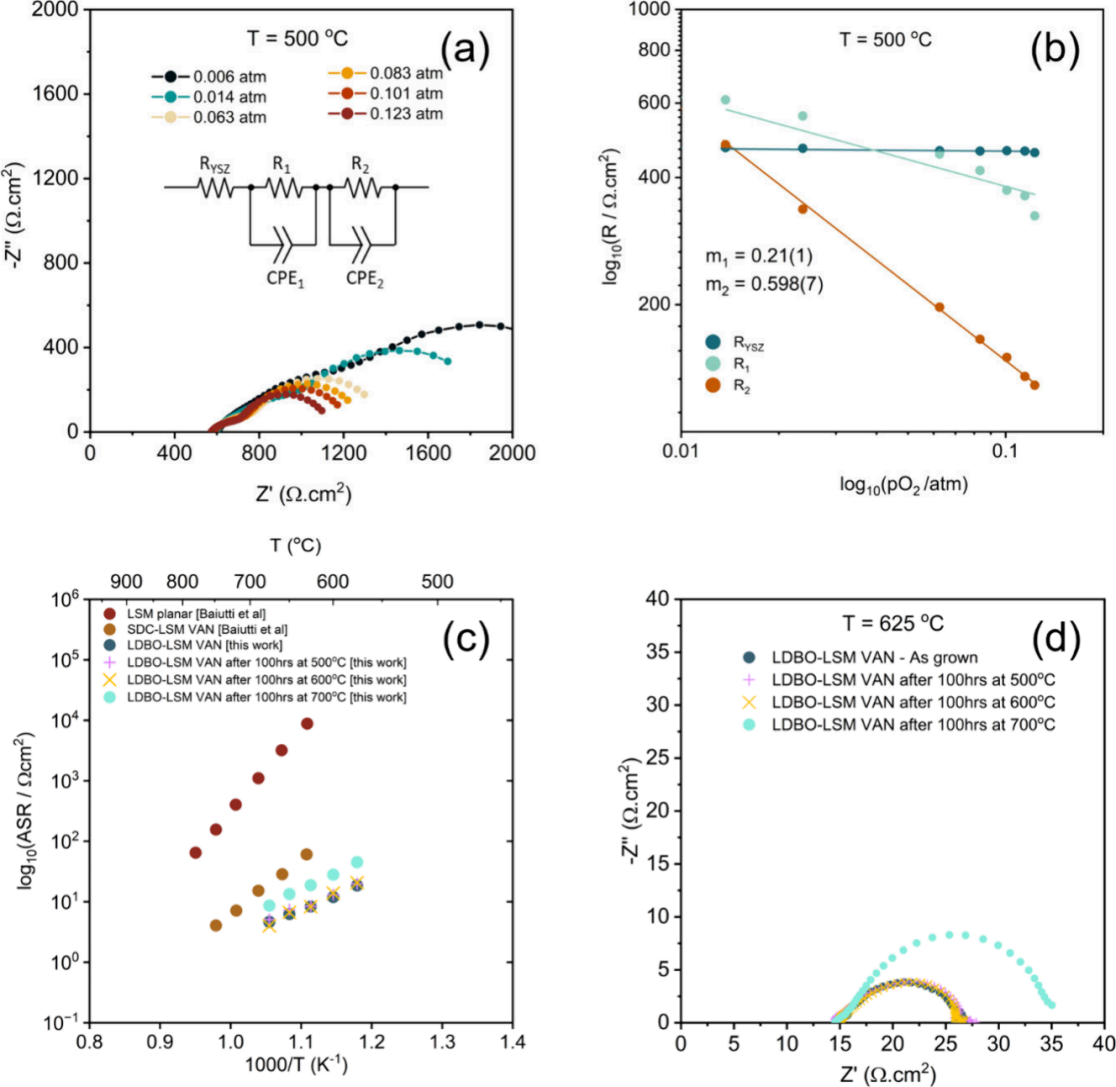
Electrical impedance spectroscopy studies of LDBO-LSM VAN films on YSZ (001) substrates. (a) Nyquist plot showing *p*O_2_ dependence of impedance features, with the circuit model inset. (b) Arrhenius plot showing *p*O_2_ dependence of *R*_YSZ_, *R*_1_, and *R*_2_ elements, with the reaction orders inset. (c) Arrhenius plot comparing ASR of LDBO-LSM VAN films (before and after degradation at 500, 600, and 700 °C for 100 h) with literature values for planar LSM and LSM-SDC VAN films from ref (1). (d) Nyquist plots measured at 625 °C of LDBO-LSM VAN films before and after degradation for 100 h at 500 °C, 600 °C, and 700 °C, respectively.

To establish the nature of the rate-limiting ORR subprocesses (adsorption, dissociation, reduction, surface diffusion to the TPB, or charge transfer), the reaction order is determined from logarithmic plots of the ASR dependence on pO_2_ (Figure 2b)_._ This is given by the equation
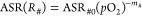
where ASR_#0_ is the pre-exponential factor and *m*_#_ is the reaction order.^22,36−39^ The two ORR processes (P_1_ and P_2_) have reaction orders of *m*_1_ = 0.21(1) and *m*_2_ = 0.598(7), respectively. Comparing these values to widely reported reaction orders of the ORR subprocesses, P_1_ is ascribed to LSM surface diffusion of the oxygen ion to the triple phase boundary (*m*_1_ ≈ 1/4) and P_2_ to reduction of the adsorbed O_ads_ to O_ads_^2–^ on LSM (*m*_2_ ≈ 1/2).^36^ For higher *p*O_2_, *R*_1_ > *R*_2_, and so we can conclude that surface diffusion (P_1_) becomes rate limiting. Below *p*O_2_ = 0.0078 atm (where *R*_1_ ≈ *R*_2_), this relation inverts (*R*_2_ > *R*_1_) so that oxygen reduction becomes rate limiting (P_2_). Crucially here all rate-limiting steps are associated with LSM ORR processes. Hence, the mass transport contribution from the intrinsically low ionic conductivity of LSM is successfully compensated in the VAN structure by the fast ionic conduction of the La-doped Bi_2_O_3_ columns and short (nm) diffusion path lengths in the 3D nanostructure, as shown schematically in Figure 3. Thus, ionic transport in the LDBO is not the limiting contribution to the total ASR of the cathode.^34^ In fact, as we later show in variable-temperature EIS measurements (Figure 2c), inclusion of LDBO significantly enhances ORR kinetics and thus reduces the total ASR relative to planar LSM (Figure 3). *R*_YSZ_ corresponds to the O^2–^ ionic resistance through the YSZ substrate (9.5 mol % Y_2_O_3_), which, as expected, shows no dependence on *p*O_2_ (*m* = 0) for this composition over the pressure range investigated.^40^

**Figure 3:**
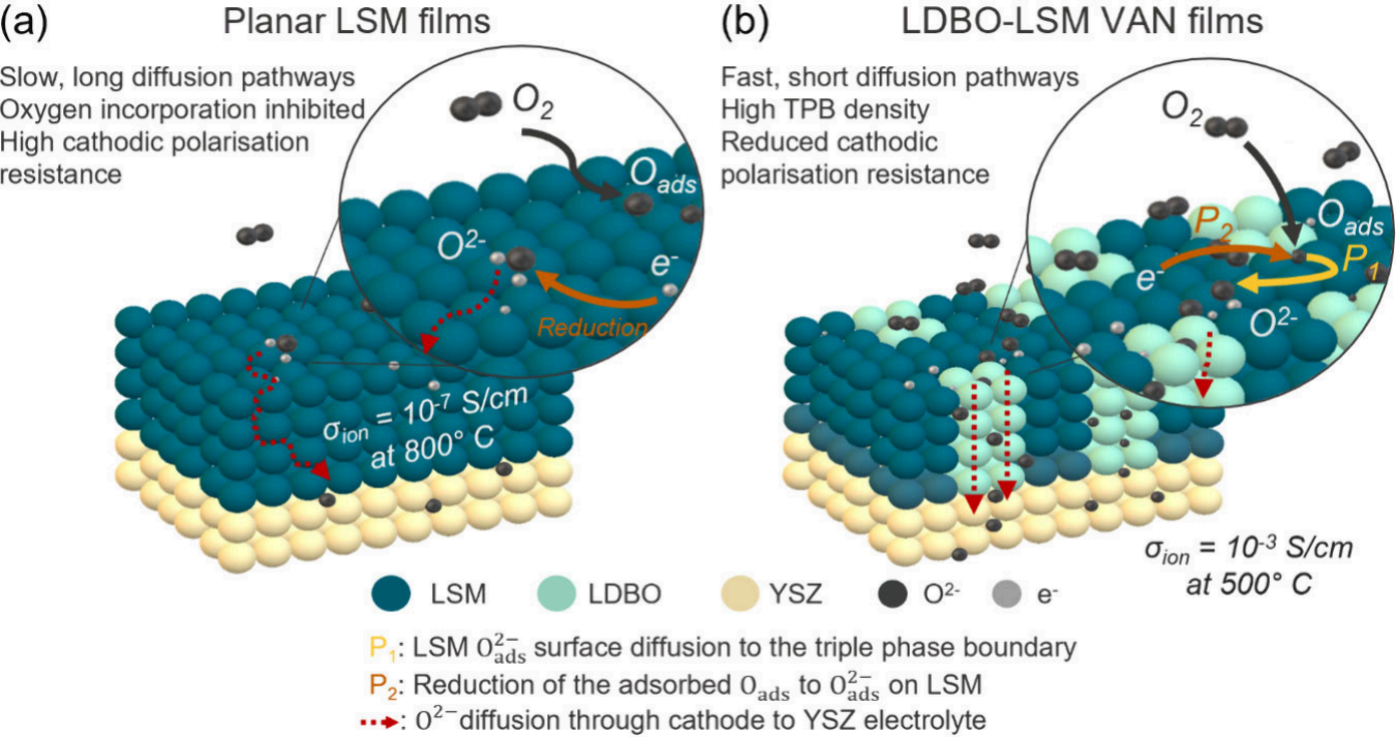
Schematic of (a) planar LSM and (b) LDBO-LSM VAN films detailing how the high ionic conducting LDBO phase can aid surface oxygen incorporation of LSM. The high density of triple phase boundaries at the LDBO-LSM VAN film surface helps facilitate a low ASR cathode.

Next, we consider the electrochemical performance of the LDBO-LSM VAN electrodes through evaluation of the ASR on a porous-Au/LDBO-LSM/YSZ/Ag system, as determined from EIS. We note here the consistency of the measurement setup with that used in the literature for the characterization of SDC-LSM films.^1^ EIS data are once again modeled by the equivalent circuit shown in Figure 2(a). Arrhenius plots (Figure 2c) reveal a low activation energy of *E*_a_ = 0.95 eV together with total ASR values (ASR = ASR_1_ + ASR_2_) of 8.3 Ω cm^2^ at 625 °C. This corresponds to a reduction of 86% compared to SDC-LSM (60.6 Ω cm^2^) and approximately 3 orders of magnitude lower than planar LSM thin films. Representative Nyquist plots in Figure 2d are consistent with the Nyquist plots for previously reported SDC-LSM VANs. Whereas, at comparable temperatures, planar LSM films are known to exhibit a Warburg diffusion response in the low-frequency regime (indicating diffusion limitations), both SDC-LSM and our LDBO-LSM VAN films exhibit a near-ideal impedance arc indicative of a purely surface reaction-limited process (Figure 2d).^1^ In essence, the improved ionic conductivity of the LDBO in the LDBO-LSM VAN films overcomes the high ionic resistance of pure LSM films (Figure 3). At the same time, the electronic carriers in the LSM enable effective oxygen reduction. Overall, a drastically reduced ASR in the VAN films results, thereby enabling lower temperature SOFC operation.

It is instructive here to consider the ASR values of the LSM-based nanocomposite thin films in reference to comparable bulk materials. In bulk materials it has been previously shown that a reduction in ASR (from 4.0 to 0.58 Ω cm^2^ at 625 °C) can be achieved by substituting SDC for yttrium-stabilized Bi_2_O_3_ (YSB), concomitant with the higher ionic conductivity of YSB.^39^ In thin films, at 625 °C we likewise observe a reduction in ASR from 60.6 Ω cm^2^ SDC-LSM VANs to 8.3 Ω cm^2^ for LDBO-LSM VANs. This is again consistent with the replacement of SDC with a higher O^2–^ ionic conducting LDBO phase (LDBO: 10^–2^ S cm^–1^ vs SDC: 10^–3^ S cm^–1^ at 500 °C, respectively^33,34^). It should be noted that subtle differences in the nanostructures of the two VAN systems may also influence the ionic transport characteristics through the film. Nevertheless, as shown in Figure 2b, ionic transport is not a rate-limiting step in this LDBO-LSM thin film. Therefore, it can be understood that the higher ionic conduction of the LDBO phase facilitates a faster rate of oxygen incorporation and therefore effectively reduces the cathodic polarization resistance, as reported previously and shown schematically in Figure 3.^39^

We also investigate the effect of thermal treatment on the LDBO-LSM VAN films to establish long-term stability characteristics. Samples were treated at 500 °C, 600 °C, and 700 °C, each for 100 h. The LDBO-LSM VAN ASR and representative Nyquist plots after each treatment are presented in Figure 2c and 2d, respectively. After treatment at 500 and 600 °C, no change in total ASR is observed, highlighting excellent stability of the material for low-temperature SOFC applications. To further explore higher temperature stability and thus to compare with planar LSM and other VAN cathodes in the literature, heat treatment was carried out in air at 700 °C for 100 h. We find the ASR increases for the LDBO-LSM VAN (Figure 2c), with an observed increase by ∼63% measured at 675 °C. This is also accompanied by a small decrease in lattice parameter for both LDBO and LSM (Supplementary Figure S3). The long-term stability of planar LSM and SDC-LSM VAN films after an equivalent heat treatment reveals a 55% increase and 72.5% decrease in ASR, respectively.^1^ In LSM, this degradation is predominantly attributed to segregation of Sr to the film surface,^1^ whereas in SDC-LSM VANs this degradation is suppressed due to the unique introduction of cerium to the LSM lattice.^1^ The degradation observed for our LDBO-LSM VANs is therefore highly consistent with the degradation anticipated for planar LSM. Thus, this highlights that VAN films are not necessarily intrinsically more stable than their planar counterparts; rather the careful pairing of matrix and columnar phases is crucial to improving thermal stability. This is an important demonstration that stability is dependent on the particular nature of the phase segregation and (any) intermixing between the two phases that results from the VAN thermodynamic self-assembly. Therefore, we highlight that to improve future VAN SOFC cathode structures, a more holistic consideration of material pairing should be considered for the intended operation temperature range.

Finally, beyond utilization as stand-alone cathodes, we note the successful deployment of SDC-LSM and YSZ-LSM films as functional interlayers in bulk anode supported SOFCs, in which the nanocomposite structure is shown to significantly enhance cell power output when incorporated between bulk electrolyte and cathode materials.^41,42^ Based on the similarity of the aforementioned VAN combinations it is highly likely that a similar strategy could be successful with our-LDBO-LSM films in the context of future SOFCs with Bi_2_O_3_-based electrolytes.

In conclusion, we demonstrate the successful growth of a La-doped Bi_2_O_3_–La_0.8_Sr_0.2_MnO_3_ (LDBO-LSM) vertically aligned nanocomposite thin film cathode grown on yttria-stabilized zirconia single-crystal substrates. This material combination enables low area specific resistance values of ∼12 Ω cm^2^ at 600 °C with exceptional long-term stability, with no degradation observed over 100 h. This corresponds to approximately 1 order of magnitude improvement over previously reported samarium-doped ceria-La_*x*_Sr_1–*x*_MnO_3_ VAN cathodes and, crucially, 3 orders of magnitude improvement over planar La_*x*_Sr_1–*x*_MnO_3_ films. Therefore, it can be concluded that the novel LDBO-LSM nanocomposite thin films presented herein represent a promising candidate material for future low-temperature (<600 °C) SOFC applications, either as a standalone cathode or as an interlayer between a bulk Bi_2_O_3_-based electrolyte and mixed Bi_2_O_3_-containing cathode.

## Supporting Information Available

Experimental methods, supporting figures (S1: HAADF-STEM EDX of LDBO-LSM VAN on YSZ (001) substrate, S2: XRD pattern of LDBO-LSM VAN on Nb-SrTiO_3_ (001) substrate, S3: XRD pattern of LDBO-LSM VAN on YSZ (001) substrate before and after thermal treatment at 700 °C) (PDF)
