# Supplementary material for: Low Area Specific Resistance La-Doped Bi2O3 Nanocomposite Thin Film Cathodes for Solid Oxide Fuel Cell Applications
Source: Nano Lett. 2024 Nov 26;24(49):15575–81. doi: 10.1021/acs.nanolett.4c03679 (PMC11639050; doi:10.1021/acs.nanolett.4c03679)
Supplement: Supplementary file 1 — nl4c03679_si_001.pdf [file nl4c03679_si_001.pdf]

Supporting Information: Low Area Specific Resistance La-doped Bi_2O_3 Nanocomposite Thin Film Cathodes for Solid Oxide Fuel Cell Applications

Adam J. Lovett[1,2]* Matthew P. Wells[1]*, Yizhi Zhang[3], Jiawei Song[3], Thomas S. Miller[2], Haiyan Wang[3] and Judith L. MacManus-Driscoll[1]*

*Joint First authors

[1]Department of Materials Science and Metallurgy, University of Cambridge, 27 Charles Babbage Road, Cambridge, United Kingdom, CB3 0FS

[2]Electrochemical Innovation Lab, Department of Chemical Engineering, University College London, Torrington Place, London, United Kingdom, WC1E 7JE

[3]School of Materials Engineering, Purdue University, 701 West Stadium Avenue, West Lafayette, Indiana, United States of America, 47907-2045

Corresponding authors: Adam J. Lovett (adam.lovett@ucl.ac.uk), Matthew P. Wells (mpw52@cam.ac.uk) and Judith L. MacManus-Driscoll (jld35@cam.ac.uk)

Experimental Methods

Pulsed Laser Deposition and Target Preparation

Target Preparation: A composite target was prepared by mixing $La_{0.8}Sr_{0.2}MnO_3$ (LSM) (Sigma Aldrich) and Bi_2O_3 (Sigma Aldrich) in a 1 : 1 ratio by volume, pelletising and sintering at 750 °C for 10 hours. A 20% wt. excess of Bi_2O_3 was added to the target compensate for bismuth loss during PLD.

Pulsed Laser Deposition: LDBO-LSM thin films were grown by pulsed laser deposition (PLD) using a 248 nm wavelength KrF laser (Lambda Physik, Inc). Films were grown on (001) oriented $(Y_2O_3)_{0.095}(ZrO_2)_{0.905}$ (YSZ) (CrysTec GmbH) and (001) oriented Nb-doped (0.5 wt%) $SrTiO_3$ (Nb-STO) substrates. Before growth, the chamber was evacuated to $7x10^{-6}$ mbar before filling to a growth pressure of 0.13 mbar pO_2 at a flow rate of 6 sccm. Films were grown with the following PLD process parameters: T_{sub} = 760 °C, fluence = 2.2 J cm^{-2}, pO_2 = 0.13 mbar, ν = 3 Hz, substrate-target distance = 45 mm. After growth, films were cooled to room temperature at 10 °C/min.

Structural Characterisation

Thin Film X-Ray Diffraction: Films were characterized with high resolution X-ray diffraction (XRD) performed on a Panalytical Empyrean vertical diffractometer using a Cu Kα X-ray radiation source with a wavelength of 1.5418 Å.

Electron Microscopy: Film microstructure was characterized with Transmission Electron Microscopy (TEM), Scanning Transmission Electron Microscopy (STEM) and STEM electron-dispersive X-ray spectroscopy (EDX) using a FEI TALOS 200X (at 200 kV) and a FEI TitanTM G2 80-200 STEM (operated at 200 kV with Cs probe corrector and ChemiSTEMTM) electron microscope. High-angle annular dark-field (HAADF) images were acquired over a collection range of 60-160 mrad.

Conductive Atomic Force Microscopy: Atomic force microscopy (AFM) measurements were carried out using a Bruker Dimensions Icon with ScanAsyst housed in an Ar-filled glovebox (< 0.5 ppm H_2O and O_2). The conductive AFM (C-AFM) mode was utilised to characterize the film topography and conductive properties with SCM-PIC-V2 AFM tips (Bruker, PtIr coated Sb (n) doped Si with nominal tip radius = 25 nm, k = 0.1 N m^{-1}¸f_0 = 10 kHz). LDBO-LSM films grown on electronically conductive Nb-$SrTiO_3$ (001) substrates were mounted onto the AFM stage with conductive Ag-paste.

Electrochemical Characterization

Variable temperature electrochemical impedance spectroscopy (EIS) measurements were performed in air between 575 and 675 °C on a Ag(bottom electrode)/YSZ(substrate)/LDBO-LSM(VAN film)/porous-Au(top electrode) system. At each temperature, the sample was left for 5 minutes to ensure that thermal equilibrium was achieved. Samples were mounted directly onto a heater block using Ag-paste. Current collection was achieved using a porous Au paste applied to the top of the films and a low impedance Ag-paste counter electrode applied to the bottom. EIS measurements were acquired using a Materials Lab XM Impedance Analyser (Ametek Inc) with a 20 mV AC voltage over a frequency range 1 Hz – 1 MHz. Film degradation was studied by annealing samples for 100 hours in air at 500, 600, and 700° C using a tube furnace (Carbolite Gero Ltd) with a ramp rate of 5° C/min during heating and cooling.

Variable oxygen partial pressure (pO_2) EIS measurements on a Ag(bottom electrode)/YSZ(substrate)/LDBO-LSM(VAN film)/Ag(top electrode) system was performed at 500 °C in a LinkAm TS1000 heating stage fitted with probe arms. The oxygen partial pressure was adjusted by mixing controlled ratios of O_2 and Ar gas, measured with a Cambridge Sensotec Rapidox 2100 oxygen analyser. The sample was left for 10 minutes after the pO_2 had stabilized to ensure atmospheric equilibrium was achieved. EIS measurements were performed using a Biologic SP200 potentiostat with a 20 mV applied AC voltage between 0.1 Hz–10 kHz. The samples were mounted on a thin sapphire slip with conductive Ag-paste (pre-set at 100 °C), which also acted as the bottom electrode. An Ag-paste top electrode (pre-set at 100 °C) was painted on the film surface, with the area measured with optical microscopy.

Supporting Figures

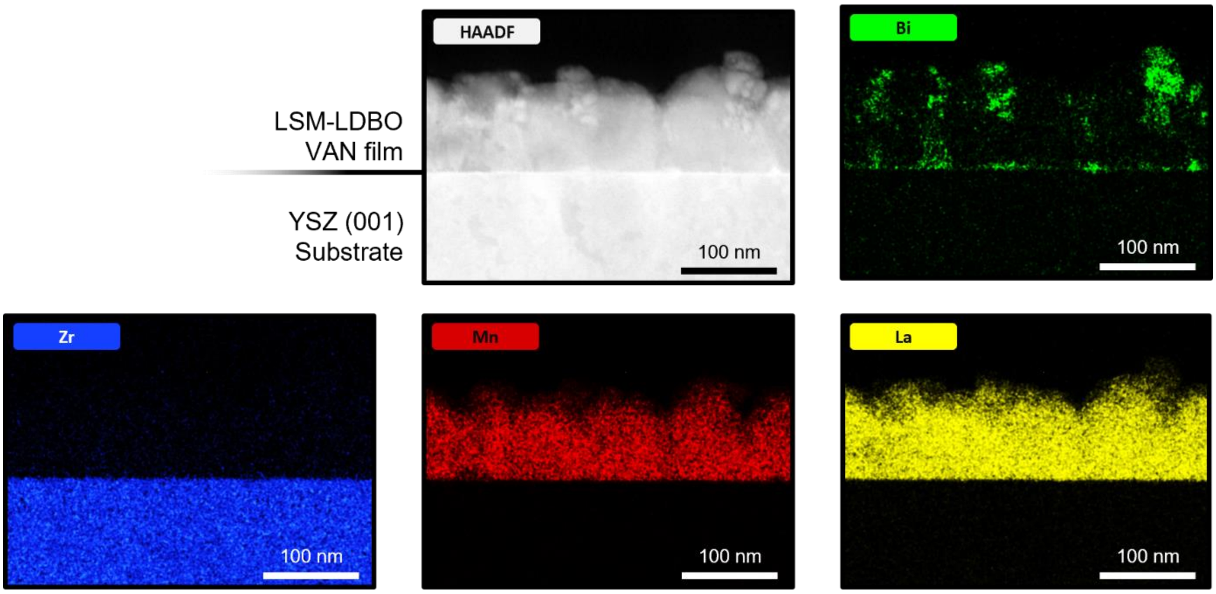

Figure S1: HAADF-STEM with EDX of a LSM-LDBO VAN film. Bismuth EDX images confirming localised Bi as part of fast ionic conducting LDBO columns.

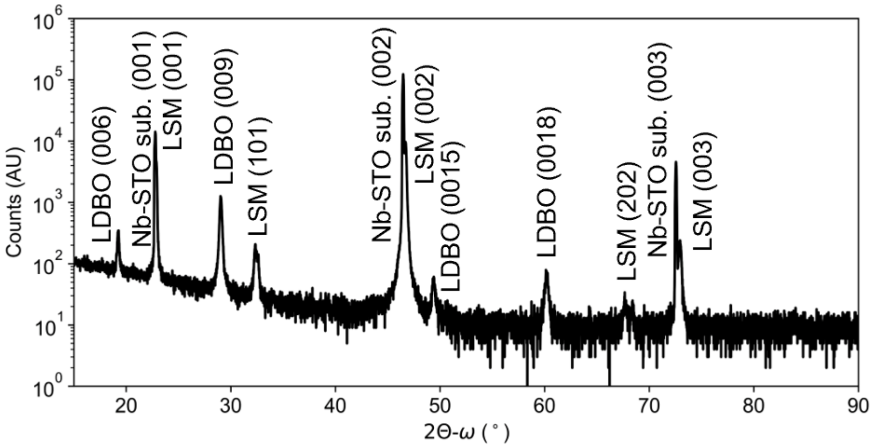

Figure S2: XRD pattern of LDBO-LSM VAN film on Nb-$SrTiO_3$ (001) substrate, indicative of epitaxial nature of both phases i.e., very sharp LDBO (00*l*) family of reflections, and both the LSM (00*l*) and LSM (*h*0*l*) *h* = *l* families. This film shares the same epitaxial relationship as films on YSZ (001) substrates (Fig. 1d).

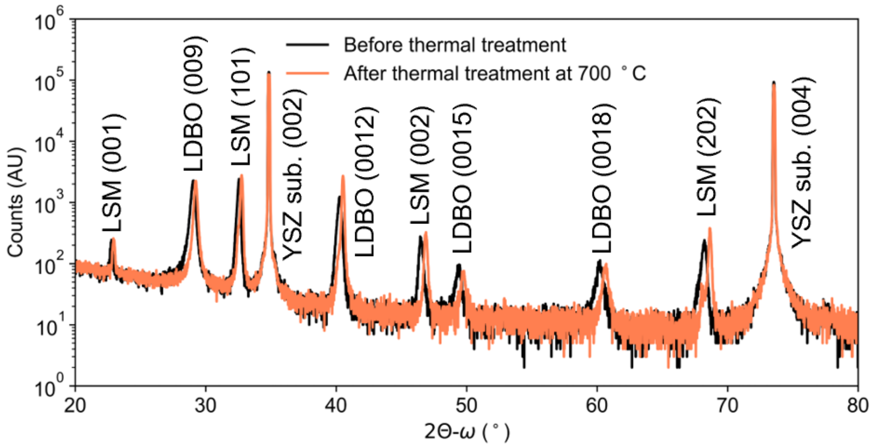

Figure S3: XRD pattern of LDBO-LSM VAN film on YSZ (001) before and after thermal treatment at 700 °C. All film reflections slightly shift to higher 2θ-ω, indicating a small decrease in lattice parameters for both LSM and LDBO post heat treatment. No additional reflections are observed, indicating no new phases form.
